# Diagnosis by Resolution: Ultrasound Follow‐Up of Neonatal Polypoid Cystitis Mimicking Botryoid Rhabdomyosarcoma

**DOI:** 10.1002/jcu.70286

**Published:** 2026-06-11

**Authors:** Davide Meneghesso, Mattia Pasquinucci, Martina Bruniera, Filippo Carletti, Alessandro Morlacco

**Affiliations:** ^1^ UOC Pediatria e Patologia Neonatale Ospedale di Bassano del Grappa Bassano Del Grappa Italy; ^2^ UOC Urologia Pediatrica Azienda Ospedale Università Padova Padova Italy; ^3^ DISCOG Università Degli Studi di Padova Padova Italy

**Keywords:** botryoid rhabdomyosarcoma, conservative management, neonatal bladder mass, polypoid cystitis, ultrasonography

## Abstract

Bladder masses in neonates are rare and often raise concern for rhabdomyosarcoma; benign inflammatory conditions may mimic malignant diseases on ultrasound. A 16‐day‐old male presented with hematuria and blood clots, no clinical symptoms. Urinalysis showed pyuria, and urine culture was positive for *Escherichia coli* . Ultrasound revealed bladder‐wall thickening and an 8‐mm polypoid lesion, suspicious for botryoid‐rhabdomyosarcoma. Given the patient's condition, a conservative approach with antibiotics and ultrasound follow‐up was adopted. At 12 days, ultrasound demonstrated complete bladder normalization. In selected stable neonates, short‐interval US follow‐up may support diagnosis through resolution, helping to distinguish inflammatory conditions from malignancy. This approach may reduce unnecessary procedures.

## Graphical abstract

In neonatal bladder masses, benign inflammatory conditions like polypoid cystitis can mimic malignancy on ultrasound. In stable neonates with evidence of infection, short‐interval ultrasound follow‐up after antibiotic therapy allows “diagnosis by resolution.” This conservative approach safely avoids unnecessary invasive procedures while maintaining oncologic vigilance.

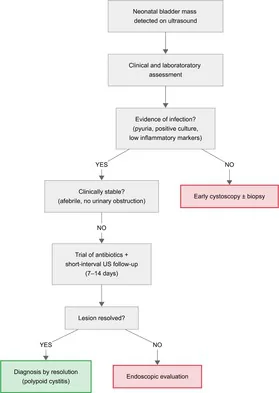

## 1 Introduction

The detection of a bladder mass in a neonate immediately raises concern for malignancy. Among these, botryoid rhabdomyosarcoma (RMS) represents the most clinically significant malignant consideration due to its aggressive course and poor prognosis. RMS has an incidence of 4.7 per million children and adolescents under 20 years of age, with genitourinary tract involvement accounting for approximately 20% of cases, predominantly in children aged 1–4 years. Its occurrence in neonates is exceptional (Martin‐Giacalone et al. 2021). However, not all tumor‐like bladder lesions are neoplastic; rare benign inflammatory conditions such as polypoid cystitis may present overlapping imaging features (Arena et al. 2015).

Polypoid cystitis is a reactive inflammatory process characterized by edematous mucosal projections, typically associated with infection or irritation. Its occurrence in neonates is extremely rare, being more commonly described in adults and older children. Because of its rarity (Arena et al. 2015) and its imaging similarity to malignant conditions (Martin‐Giacalone et al. 2021), tumor‐like bladder lesions in neonates often prompt early invasive diagnostic procedures.

We report a neonatal case of a bladder lesion highly suspicious for RMS on ultrasound that is completely resolved with conservative management, highlighting the potential role of short‐term ultrasound follow‐up in avoiding unnecessary invasive interventions.

## 2 Case Report

A 16‐day‐old male neonate was referred for gross hematuria with blood clots. Pregnancy and delivery were uneventful. At presentation, the infant appeared well, afebrile, and hemodynamically stable, without signs of systemic illness.

Dipstick urinalysis revealed hematuria (3+) and leukocyturia (3+). Urine microscopy showed marked pyuria (1039 WBC/μL) with a relatively low erythrocyte count, likely reflecting erythrocyte lysis in diluted urine (specific gravity 1.004). Blood test demonstrated minimal inflammatory response (CRP 0.81 mg/dL; procalcitonin 0.08 μg/L). Catheterized urine culture isolated *Escherichia coli* (50 000 CFU/mL), susceptible to amoxicillin–clavulanate.

Renal and bladder ultrasound demonstrated diffuse bladder wall thickening and an 8‐mm polypoid lesion adjacent to the right ureteral orifice (Figures 1 and 2). Both kidneys appeared normal. The lesion showed a lobulated intraluminal appearance arising from the bladder wall, raising concern for botryoid RMS. Differential diagnoses included RMS, inflammatory pseudotumor, and polypoid cystitis (Table 1).

**FIGURE 1:**
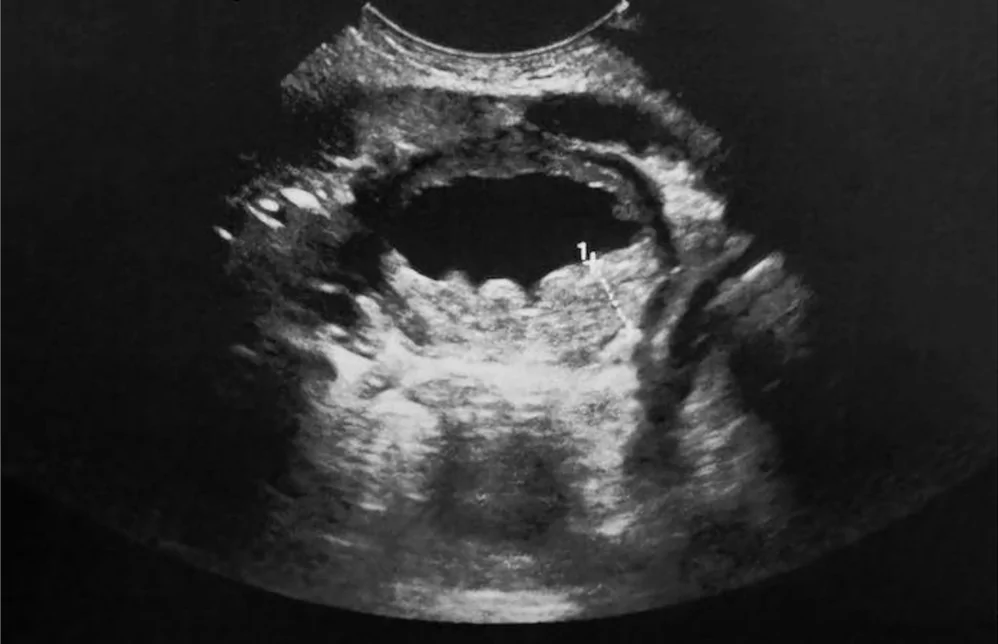
Initial ultrasound scan showing diffuse, irregular bladder wall thickening and 8‐mm focal polypoid lesion near the right ureteral orifice, mimicking a rhabdomyosarcoma.

**FIGURE 2:**
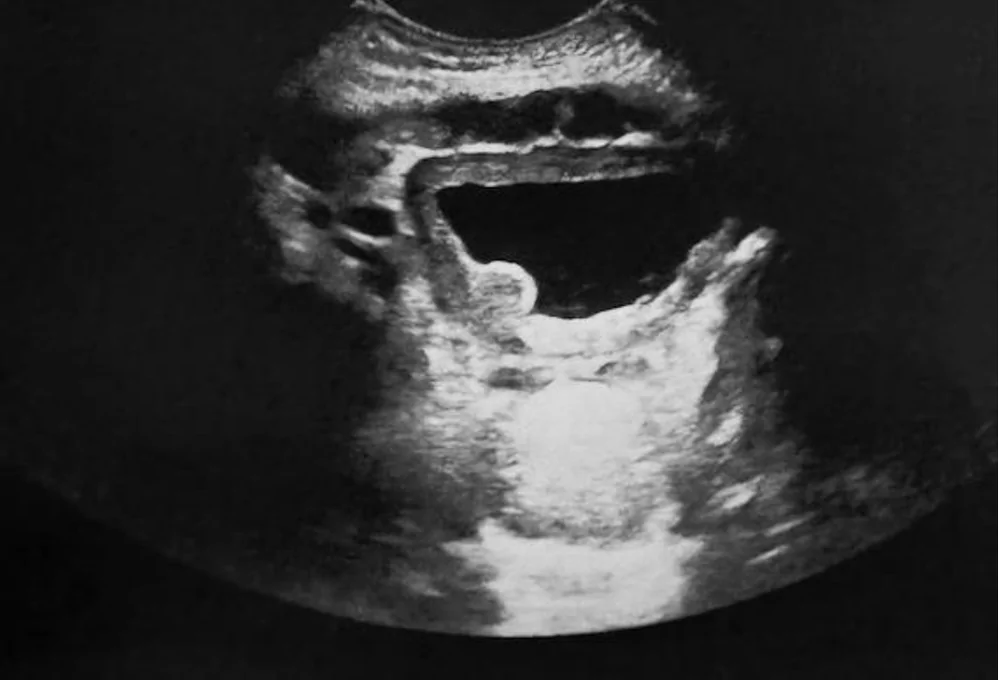
Initial ultrasound scan showing diffuse, irregular bladder wall thickening and 8‐mm focal polypoid lesion near the right ureteral orifice, mimicking a rhabdomyosarcoma.

**TABLE 1:** Differential diagnosis of neonatal bladder masses.

Condition	Typical ultrasound appearance	Clinical clues	Key distinguishing features	Need for urgent endoscopic evaluation
Bladder rhabdomyosarcoma	Polypoid or grape‐like intraluminal mass, often lobulated or multiple	Hematuria, possible urinary obstruction, usually no infection	Persistent or progressive lesion; malignant potential	Yes—histological confirmation required
Polypoid cystitis	Focal or diffuse polypoid mucosal thickening	Hematuria, pyuria, often associated with UTI or instrumentation	Rapid regression with antibiotic therapy or removal of irritation	Usually no, if clinically stable and rapidly resolving
Inflammatory pseudotumor (myofibroblastic tumor)	Solid heterogeneous lesion arising from bladder wall	Hematuria, occasional systemic inflammation	May show local aggressiveness; diagnosis uncertain on imaging	Often yes—biopsy frequently required
Bladder hematoma	Echogenic intravesical mass without vascularity	Recent trauma, catheterization, or coagulopathy	Mobility or spontaneous resolution on follow‐up imaging	No, unless persistent or obstructive
Ureterocele (prolapsing intravesical)	Thin‐walled cystic lesion near ureteral orifice	Associated hydronephrosis or duplex system	Cystic appearance with ureteral continuity	Not usually urgent; based on obstruction or infection
Congenital bladder polyp (rare)	Solitary intraluminal polypoid lesion	Intermittent hematuria or voiding symptoms	Benign but persistent lesion	Case‐dependent, if symptomatic or persistent
Blood clots	Mobile echogenic nonvascular material	Gross hematuria	Shape and location change on serial scans	No

Given the infant's excellent clinical condition, absence of systemic inflammation, and microbiological evidence of urinary tract infection, a multidisciplinary decision favored conservative management. The patient was admitted for close monitoring and treated with oral amoxicillin–clavulanate. Cystoscopy and biopsy were initially deferred in favor of conservative treatment to avoid potential iatrogenic injury to the neonatal urinary tract.

The clinical course was uneventful, with progressive resolution of hematuria. Follow‐up ultrasound at 12 days demonstrated complete disappearance of the lesion and normalization of bladder wall thickness (Figure 3), supporting an inflammatory etiology consistent with polypoid cystitis.

**FIGURE 3:**
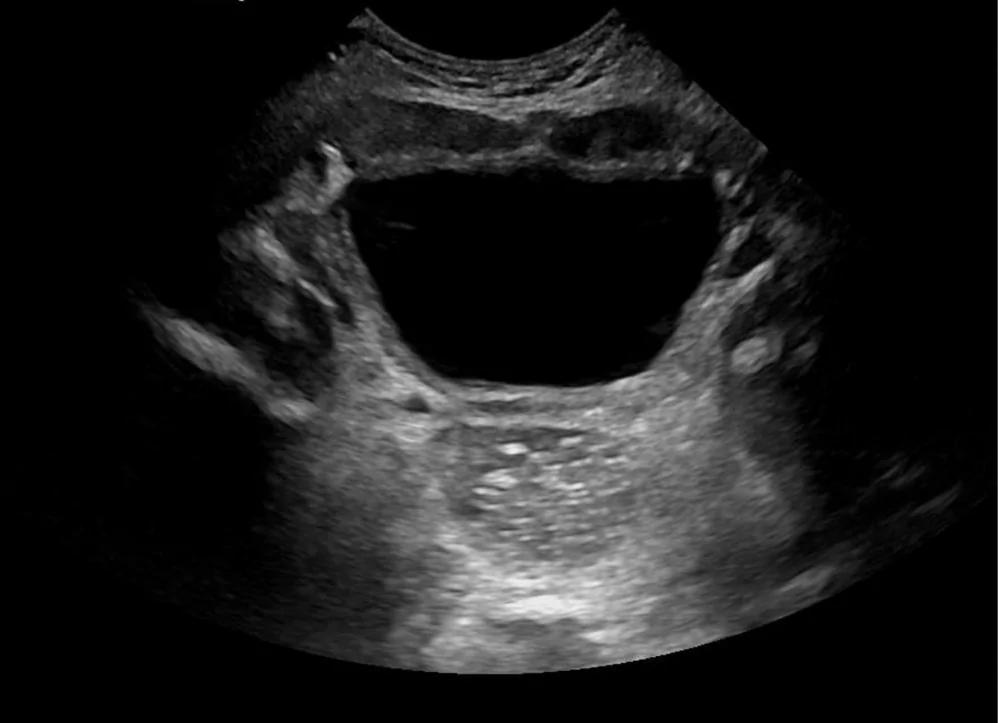
Follow‐up scan after conservative treatment, demonstrating complete resolution of the polypoid mass and normalization of the bladder wall thickness.

## 3 Discussion

Neonatal bladder masses are rare and represent a diagnostic challenge in pediatric urology. Given that RMS is the primary malignant diagnosis to exclude, tumor‐like intravesical lesions often prompt easily invasive evaluation. However, inflammatory conditions may closely mimic neoplastic lesions on ultrasound, creating a potential risk of overtreatment (Arena et al. 2015; Shelmerdine et al. 2017).

In clinically stable patients, careful assessment of inflammatory markers, microbiological results, and general condition may help guide the timing of further diagnostic interventions, balancing the risk of delayed diagnosis of malignancy against unnecessary invasive procedures.

Polypoid cystitis is a reactive inflammatory condition only exceptionally described in neonates. Although data in this population are limited, available pediatric literature suggests that inflammatory tumor‐like lesions may regress following appropriate treatment of underlying causes. In our case, several factors supported a conservative approach: clinical stability, absence of fever or systemic inflammation, positive urine culture, and normal upper urinary tract anatomy. The rapid and complete resolution observed on short‐interval ultrasound follow‐up strongly argued against malignancy (Rosenberg et al. 1994; Shelmerdine et al. 2017).

Avoiding immediate endoscopic evaluation in selected patients may reduce the risk of iatrogenic complications, including urethral trauma or bladder injury, which would be particularly relevant in neonates. A carefully monitored period of observation, combined with targeted antibiotic therapy and early ultrasound reassessment, may represent a balanced and safe diagnostic strategy in equivocal cases (Figure 4) (Rosenberg et al. 1994).

**FIGURE 4:**
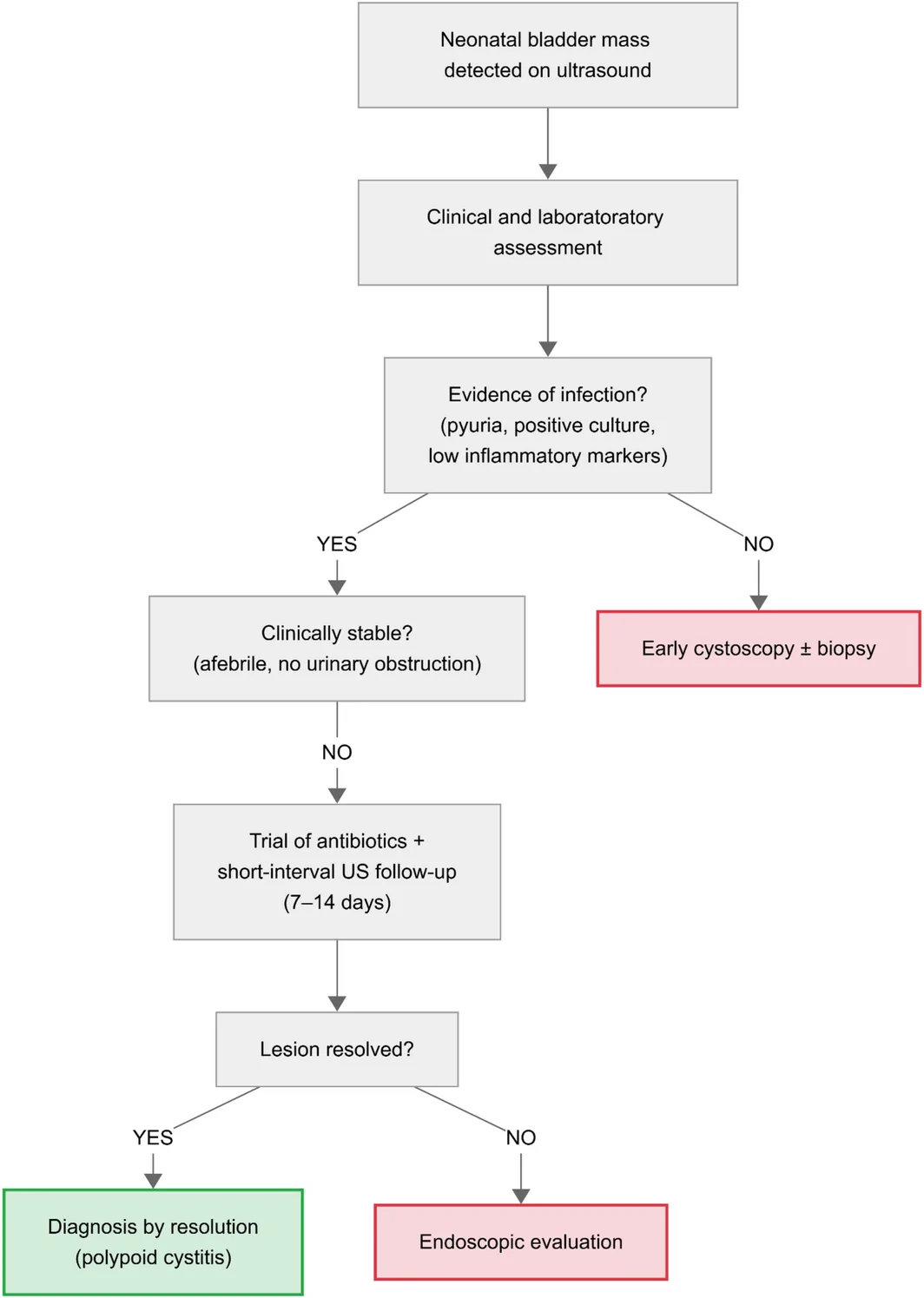
Clinical take‐home algorithm.

Persistence, growth, or recurrence of the lesion should be treated with endoscopic and histological evaluation.

## 4 Conclusion

This case highlights that, in carefully selected clinically stable neonates, short‐interval ultrasound follow‐up may allow diagnosis by resolution, safely avoiding unnecessary invasive procedures while maintaining oncologic vigilance. Strict adherence to clinical, laboratory, and imaging selection criteria remains essential, and further multicenter case collections are needed to validate this approach and establish standardized management guidelines.

## Funding

The authors have nothing to report.

## Ethics Statement

Ethical approval was waived by the local institutional review board as this is a single case report involving standard clinical practice.

## Consent

Informed consent was obtained from the patient's legal guardians for the publication of this case report and accompanying images.

## Conflicts of Interest

The authors declare no conflicts of interest.

## Data Availability

The data that supports the findings of this study are available from the corresponding author upon reasonable request.
